# Imaging of cortical oxygen tension and blood flow following targeted photothrombotic stroke

**DOI:** 10.1117/1.NPh.5.3.035003

**Published:** 2018-07-27

**Authors:** Colin T. Sullender, Andrew E. Mark, Taylor A. Clark, Tatiana V. Esipova, Sergei A. Vinogradov, Theresa A. Jones, Andrew K. Dunn

**Affiliations:** a University of Texas at Austin, Department of Biomedical Engineering, Austin, Texas, United States; b University of Texas at Austin, Department of Psychology, Austin, Texas, United States; c University of Texas at Austin, Institute for Neuroscience, Austin, Texas, United States; d University of Pennsylvania, Department of Biochemistry and Biophysics, Philadelphia, Pennsylvania, United States

**Keywords:** laser speckle contrast imaging, oxygen tension, phosphorescence quenching, photothrombosis, ischemic stroke, imaging system

## Abstract

We present a dual-modality imaging system combining laser speckle contrast imaging and oxygen-dependent quenching of phosphorescence to simultaneously map cortical blood flow and oxygen tension (${\mathrm{pO}}_{2}$) in mice. Phosphorescence signal localization is achieved through the use of a digital micromirror device (DMD) that allows for selective excitation of arbitrary regions of interest. By targeting both excitation maxima of the oxygen-sensitive Oxyphor PtG4, we are able to examine the effects of excitation wavelength on the measured phosphorescence lifetime. We demonstrate the ability to measure the differences in ${\mathrm{pO}}_{2}$ between arteries and veins and large changes during a hyperoxic challenge. We dynamically monitor blood flow and ${\mathrm{pO}}_{2}$ during DMD-targeted photothrombotic occlusion of an arteriole and highlight the presence of an ischemia-induced depolarization. Chronic tracking of the ischemic lesion over eight days revealed a rapid recovery, with the targeted vessel fully reperfusing and ${\mathrm{pO}}_{2}$ returning to baseline values within five days. This system has broad applications for studying the acute and chronic pathophysiology of ischemic stroke and other vascular diseases of the brain.

## 1 Introduction

The measurement of blood flow and oxygen tension in cerebral vasculature is vital for the study of many physiological and pathophysiological conditions in the brain. Laser speckle contrast imaging (LSCI) is a well-established technique for full-field optical imaging of cortical blood flow.^1^^–^^4^ *In vivo* measurements of molecular oxygen have historically been made using highly invasive Clarke electrodes that are limited to point measurements outside the vascular lumen.^5^^–^^7^ Magnetic resonance techniques allow for noninvasive imaging of hemoglobin saturation, but suffer from low spatial resolutions and can only be correlated with free oxygen in the blood.^7^^–^^11^ Oxygen-sensitive porphyrin probes allow for noninvasive, highly sensitive optical oxygenation measurements based on phosphorescence quenching.^12^ While an injection of the probe is required, absolute oxygen tension (${\mathrm{pO}}_{2}$) can be directly calculated from the lifetime of the measured phosphorescence decay.

To spatially resolve the phosphorescent signal, most lifetime imaging systems utilize intensified exposure-gated cameras^13^^,^^14^ or laser scanning systems,^15^^,^^16^ both of which have limitations.^17^ Cameras suffer from poor spatial resolution because of light scattering in tissue while scanning systems lack temporal resolution because each spatial location requires many repeated measurements. The use of a digital micromirror device (DMD) as a spatial light modulator was proposed to overcome these limitations.^18^ Rather than producing a full image, arbitrary regions of interest could be sequentially targeted for selective excitation. By constraining the phosphorescent signal to only the targeted region, a point detector could be used for acquiring spatially resolved lifetime data with high sensitivity and temporal resolution.

In this paper, we present an update to the system by Ponticorvo and Dunn^18^ that utilizes a more robust porphyrin probe^19^ and offers higher spatiotemporal resolutions for both LSCI and oxygen-dependent quenching of phosphorescence. Using both of the phosphorescent probe’s excitation maxima, we highlight wavelength-dependent differences in measured decay lifetimes. We demonstrate the ability to detect static variations in ${\mathrm{pO}}_{2}$ between different types of vasculature and the dynamic monitoring of blood flow and ${\mathrm{pO}}_{2}$ during DMD-targeted photothrombotic stroke. We also demonstrate chronic imaging by tracking the response to an ischemic event over several days. This system allows for acute and chronic imaging of relative blood flow and oxygen tension and has broad applications for both basic neuroscience and neuropathophysiology.

## 2 Methods

### 2.1 Imaging Instrumentation

**Fig. 1:**
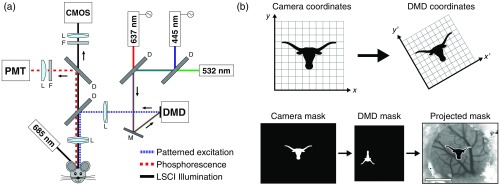
(a) Schematic of the imaging system. Three diode lasers (445, 532, and 637 nm) are coaligned and coupled with the DMD to provide structured illumination onto a cranial window. A separate 685-nm laser provides oblique illumination for LSCI. A pair of dichroic beamsplitters separate phosphorescence from scattered LSCI laser light for detection. (b) Example of the linear image transformation applied to binary masks for DMD patterning and the resulting projection as imaged using LSCI (Scale bar=1  mm).

A schematic of the imaging system is shown in Fig. 1(a). LSCI was performed using a 685-nm laser diode (50 mW, HL6750MG, Thorlabs) illuminating the craniotomy at an oblique angle. The excitation and emission spectra of the phosphorescent probe dictated dichroic beamsplitter cutoff wavelengths and limited the options for the near-infrared laser diode wavelength. The scattered light was relayed to a CMOS camera (acA1300-60 gmNIR, 1280×1024  pixels, Basler AG) with 2× magnification for a field of view of 3.5  mm×2.8  mm. Images were acquired using custom software written in C++ at 60 frames per second with a 5-ms exposure time.

Simultaneously, laser light patterned by the DMD was delivered to the craniotomy to selectively excite the phosphorescent probe. Wavelength has been shown to significantly affect the penetration depth and sampled volume of measurements through surface vasculature,^20^ so two different lasers at 445 (200 mW, AixiZ) and 637 nm (250 mW, HL6388MG, Thorlabs) were selected to target the Soret and Q band excitation maxima of the dye, respectively.^19^ Each laser was used independently to collect lifetime measurements from identical regions of interest for comparison. The 445-nm laser was gated using an acousto-optic modulator (23080-2-LTD, Neos Technologies) while the 637-nm laser was directly gated via its driver (LDD400-1P, Wavelength Electronics). Both lasers were gated to produce 20-μs pulses of light for time-domain lifetime measurements with a 6% duty cycle and ∼3-kHz repetition rate using the National Instruments FlexRIO field programmable gate array (FPGA) (NI-5781R) for waveform generation.

The modulated laser light was relayed to a DMD for projection onto the craniotomy. A DMD is an optical semiconductor device that consists of a two-dimensional array of thousands of individually addressable mirrors that can be tilted to spatially modulate light. The DMD allows for the localization of phosphorescence measurements while maintaining the high sensitivity of using a point detector.^18^ A DLP LightCrafter Evaluation Module (Texas Instruments) was modified to expose the bare DMD (DLP3000, 608×684  pixels, 7.6-μm pitch) for illumination. The projected DMD pattern was coregistered with the LSCI camera via an affine image transformation [Fig. 1(b)]. This allowed for the selection of arbitrarily shaped regions of interest using speckle contrast imagery for guidance. The resulting binary masks were then transformed into DMD coordinate space and uploaded onto the device via its USB-based application programming interface. Each pattern was then projected sequentially onto the exposed brain tissue to selectively excite the phosphorescent probe for ${\mathrm{pO}}_{2}$ measurements.

The emitted phosphorescence was separated from the excitation light and LSCI laser using a pair of dichroic beamsplitters (650 nm, ZT640rdc, Chroma Technology and 750 nm, FF750-SDi02, Semrock) and a bandpass filter (775±26  nm, 84 to 106, Edmund Optics) and relayed to a photomultiplier tube for detection (H7422P-50, Hamamatsu Photonics). The analog signal was amplified (C9999, Hamamatsu Photonics), digitized at 100 MHz, and accumulated by the FPGA for averaging, after which it was transferred to the host computer and written to file. The maximum pattern projection rate and temporal resolution of the ${\mathrm{pO}}_{2}$ measurements were limited by the strength of the phosphorescent signal. The projected pattern size, dye concentration, and excitation laser power all influenced the resulting signal intensity and the necessary averaging of phosphorescent decays. To balance the signal-to-noise ratio and speed, patterns were displayed at 10 Hz with 200 decays collected during each projection. While faster pattern rates were possible (e.g., 50 Hz with 40 decays averaged), the reduction in averaging negatively affected the quality of the recorded decays.

### 2.2 Animal Preparation

Mice (CD-1, male, 25 to 30 g, Charles River) were anesthetized with medical air vaporized isoflurane (2%) via nose-cone inhalation. Body temperature was maintained at 37°C with a feedback heating pad (DC Temperature Controller, Future Health Concepts). Arterial oxygen saturation, heart rate, and breath rate were monitored via pulse oximetry (MouseOx, Starr Life Sciences). After induction, mice were placed supine in a stereotaxic frame (Narishige Scientific Instrument Lab) and administered carprofen (5  mg/kg, subcutaneous) and dexamethasone (2  mg/kg, intramuscular) to reduce inflammation of the brain during the craniotomy procedure. The scalp was shaved and resected to expose skull between the bregma and lambda cranial coordinates. A thin layer of cyanoacrylate (Vetbond Tissue Adhesive, 3M) was applied to the exposed skull to facilitate the adhesion of dental cement during a later step. A 3-mm-diameter portion of the skull over the frontoparietal cortex was removed with a dental drill (Ideal Microdrill, 0.8-mm burr, Fine Science Tools) while leaving the dura intact. The craniotomy was performed under regular perfusion of artificial cerebrospinal fluid to protect the brain from overheating. A 5-mm round cover glass (#1.5, World Precision Instruments) was placed over the exposed brain, and a dental cement mixture was deposited along the perimeter, bonding it to the surrounding skull. This process created a sterile, air-tight seal around the craniotomy and allowed for restoration of intracranial pressure. A layer of cyanoacrylate was applied over the dental cement to further seal the cranial window. The medial and anterior edges of the window were ∼2  mm rostral to bregma and 0.5 mm lateral to midline. Animals were allowed to recover from anesthesia and monitored for cranial window integrity and normal behavior for two weeks prior to imaging. All imaging sessions were conducted using medical air with 1.5% vaporized isoflurane. The Institutional Animal Care and Use Committee at The University of Texas at Austin approved all the experiments.

### 2.3 Laser Speckle Contrast Image Analysis

The raw images captured by the camera were converted to speckle contrast images using Eq. (1), where speckle contrast (K) is defined as the ratio of the standard deviation ($\sigma_{s}$) to the mean intensity (⟨I⟩) within a small region of the image. The full speckle contrast image was calculated using a 7×7-pixel sliding window centered at every pixel of the raw image and was computed, displayed, and saved in real time using an efficient processing algorithm^21^

$$K=\frac{\sigma_{s}}{\left\langle I\right\rangle }.$$ (1)

During postprocessing, speckle contrast images were averaged together (n=45) and converted to inverse correlation time ($1/\tau_{c}$) images to provide a more quantitative measure of blood flow.^1^ The observed speckle contrast (K) was fitted for its corresponding correlation time ($\tau_{c}$) at each pixel using Eq. (2), where $x=T/\tau_{c}$.^22^ T is the camera exposure duration (5 ms) and β is an instrumentation factor that accounts for speckle sampling, polarization, and coherence effects

$$K(T,\tau_{c})={\left(\beta \frac{e^{-2x}-1+2x}{2x^{2}}\right)}^{1/2}.$$ (2)

Each inverse correlation time image was then baselined against the first frame to calculate an estimate of relative change in blood flow ($\mathrm{rCFB}=\tau_{c,\text{initial}}/\tau_{c}$).^23^ Because β is a property of the instrumentation and should not vary throughout the course of an experiment, it was assumed β=1. The same regions of interest defined for DMD structured illumination were used to calculate timecourses of the relative change in flow.

### 2.4 Oxygen Tension Measurements

**Fig. 2:**
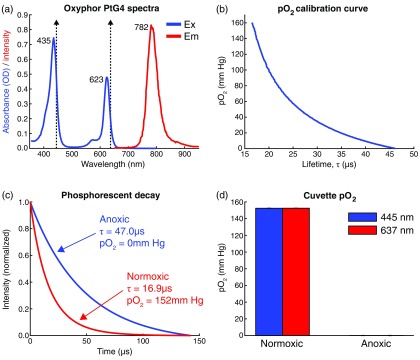
(a) Excitation and emission spectra for Oxyphor PtG4. Dashed vertical lines indicate excitation lasers at 445 nm and 637 nm. (b) Calibration curve relating ${\mathrm{pO}}_{2}$ to the measured phosphorescence lifetime (τ) under physiological conditions (37°C, pH 7.2). For τ<16  μs, Stern–Volmer kinetics were assumed with $k_{q}=291.014\;\mathrm{mm}\;{\mathrm{Hg}}^{-1}\;s^{-1}$ and $\tau_{0}=47\;\mu s$. (c) Averaged phosphorescent decay curves (n=200) in anoxic and normoxic cuvette environments with fitted lifetimes and their corresponding ${\mathrm{pO}}_{2}$ values. (d) Excitation wavelength does not affect measured ${\mathrm{pO}}_{2}$.

Oxyphor PtG4 is an oxygen-sensitive dendritic probe that contains platinum(II)-meso-tetra-(3,5-dicarboxyphenyl)tetrabenzoporphyrin as the phosphorescent core^19^^,^^24^ and has been effectively used to measure absolute oxygen tension using phosphorescence quenching.^25^^,^^26^ It has two excitation maxima near 435 and 623 nm and an emission maximum at 782 nm [Fig. 2(a)]. Oxyphor PtG4 is highly soluble in an aqueous environment without requiring the presence of environmental albumin for stabilization.^19^ For *in vivo* measurements, Oxyphor PtG4 was introduced systemically via retro-orbital injection into the venous sinus for a target blood plasma concentration of 5  μM. Phosphorescence lifetime measurements were made in the time domain using the structured pulsed excitation light paradigm described above. The instrument response was accounted for by introducing a 2-μs temporal offset to the phosphorescent signal [I(t)] prior to fitting for the decay lifetime (τ) as

$$I(t)=A+Be^{-t/\tau }.$$ (3)

A calibration curve based on Stern–Volmer kinetics^27^^,^^28^ was used to convert the measured lifetime (τ) to the absolute ${\mathrm{pO}}_{2}$. The calibration of Oxyphor PtG4 under physiological conditions is shown in Fig. 2(b). The unquenched lifetime is 47  μs in an oxygen-free environment. Figure 2(c) shows phosphorescent decay curves and their corresponding ${\mathrm{pO}}_{2}$ values within anoxic and normoxic cuvette environments with 10-μM Oxyphor PtG4. Anoxia was established using the enzymatic reaction between glucose and glucose oxidase to scavenge oxygen from a sealed cuvette.^29^ Lifetime measurements in cuvette samples did not vary with excitation wavelength [Fig. 2(d)].

### 2.5 Hyperoxic Challenge

Detection of systemic changes in oxygen tension was demonstrated by subjecting mice to a hyperoxic challenge. The oxygen fraction of inspired air under anesthesia was increased from 21% (normoxia) to 100% and then decreased back to normoxia for recovery. Hyperoxia was maintained for 5 min, and vascular ${\mathrm{pO}}_{2}$ measurements were acquired at the end of each stage. Pulse oximetry was used to monitor the status of the animal throughout the hyperoxic challenge.

### 2.6 Targeted Photothrombosis

The DMD was also used to induce arbitrarily shaped photothrombotic occlusions in the cortical vasculature using rose Bengal. Rose Bengal is a fast-clearing photothrombotic agent that photochemically triggers localized clot formation upon irradiation with green light.^30^^–^^32^ An additional 532-nm laser (200 mW, AixiZ) was coaligned with the two modulated lasers and relayed to the DMD. Structured illumination with the DMD allows for the selective targeting of individual vessels for occlusion while minimizing exposure in the surrounding parenchyma. Rose Bengal was injected intravenously (50  μL, 15  mg/mL) and the target vessels exposed to DMD-patterned green light for 5 to 10 min. Descending arterioles were the primary targets because they serve as bottlenecks in the cortical oxygen supply.^33^ Oxygen tension measurements were restricted when performing photothrombosis to the region being targeted for occlusion. LSCI was used to monitor clot formation within the targeted area and control the progression of the occlusion.

## 3 Results

### 3.1 Excitation Wavelength Dependence of Measured Oxygen Tension

**Fig. 3:**
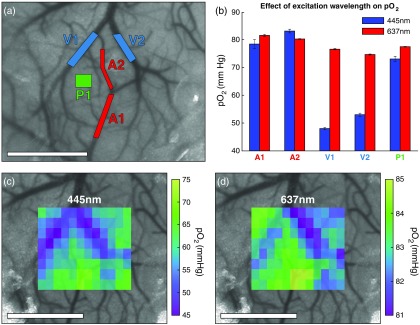
(a) Speckle contrast image of cortical flow overlaid with regions targeted for ${\mathrm{pO}}_{2}$ measurements. Two descending arterioles (A1 and A2), two veins (V1 and V2), and one parenchyma region (P1) were examined. The projected patterns ranged between 0.014 and $0.046\;{\mathrm{mm}}^{2}$ in area. (b) ${\mathrm{pO}}_{2}$ measurements within the targeted regions conducted using both 445- and 637-nm excitation of Oxyphor PtG4 (mean ± sd). (c) 445 nm and (d) 637 nm ${\mathrm{pO}}_{2}$ maps produced using tiled excitation patterns covering a 1.2- × 1.0-mm area. Each individual tile has a projected area of $0.012\;{\mathrm{mm}}^{2}$. Scale bars=1  mm.

Static oxygen tension measurements in the vasculature of the mouse cortex are shown in Fig. 3. Five different regions, including two arterioles, two veins, and an area of unresolvable vasculature (parenchyma), were targeted for selective illumination using both the 445- and 637-nm excitation lasers [Fig. 3(a)]. The measured ${\mathrm{pO}}_{2}$ within each region aligns well with physiological expectations as both arterioles have higher ${\mathrm{pO}}_{2}$ than the venous or parenchymal areas [Fig. 3(b)]. The effects of wavelength can be seen as 445-nm excitation resulted in a broader range of oxygen tension values (48 to 83 mm Hg) compared with 637-nm excitation (75 to 82 mm Hg). Despite the differences in absolute value, the trend of arterioles having greater ${\mathrm{pO}}_{2}$ than parenchyma, which in turn has greater ${\mathrm{pO}}_{2}$ than veins, exists for both excitation wavelengths. As shown previously in Fig. 2(d), no differences in ${\mathrm{pO}}_{2}$ were observed between the two wavelengths in cuvette samples.

To obtain a more comprehensive look at vascular oxygenation within the LSCI field of view, an array of 12×8 rectangular tiles was sequentially projected. The tiles were displayed at 10 Hz with 200 decays averaged per pattern for a total acquisition time of 9.6 s. The resulting ${\mathrm{pO}}_{2}$ maps [Figs. 3(c) and 3(d)] coarsely follow the visible surface vasculature. As expected, the large branching vein has lower ${\mathrm{pO}}_{2}$ values compared with the arteriole approaching from the bottom or the surrounding parenchyma. The 445-nm excitation again resulted in a wider range of ${\mathrm{pO}}_{2}$ values (45 to 85 mm Hg) compared with 637-nm excitation (81 to 85 mm Hg).

### 3.2 Hyperoxic Challenge

**Fig. 4:**
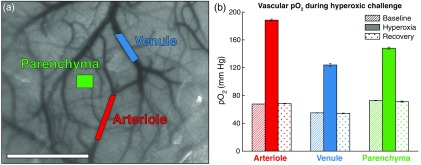
(a) Speckle contrast image depicting three regions (arteriole, venule, and parenchyma) targeted for 445-nm ${\mathrm{pO}}_{2}$ measurements during a hyperoxic challenge. (b) Static ${\mathrm{pO}}_{2}$ during baseline, hyperoxic, and recovery stages for each of the targeted vessels (mean ± sd). Scale bar=1  mm.

The ability to detect changes in neurovascular oxygen tension was tested using a hyperoxic challenge as shown in Fig. 4. Three regions covering an arteriole, venule, and parenchyma were targeted for selective 445-nm illumination [Fig. 4(a)] as the oxygen fraction was temporarily increased. Measurements were taken during each stage of the challenge and detected a large increase in ${\mathrm{pO}}_{2}$ during the hyperoxic state across all three regions [Fig. 4(b)] with the arteriole experiencing the largest net increase. The ${\mathrm{pO}}_{2}$ remained slightly elevated above baseline values several minutes later during the posthyperoxia recovery stage.

### 3.3 Oxygen Tension and Blood Flow during Photothrombosis

**Fig. 5:**
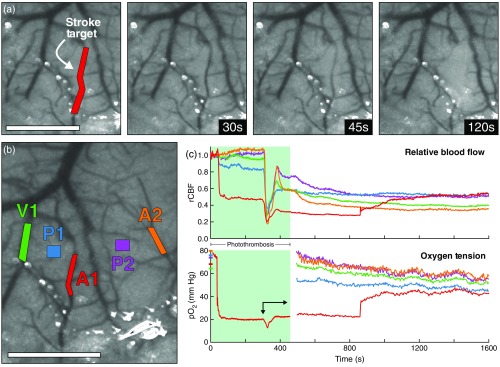
(a) Speckle contrast images depicting the occlusion of a descending arteriole using DMD-targeted photothrombosis (Video 1, MPEG4, 26.9 MB [URL: https://doi.org/10.1117/1.NPh.5.3.035003.1]). The red overlay indicates the $0.09\text{-}{\mathrm{mm}}^{2}$ region simultaneously illuminated for occlusion and ${\mathrm{pO}}_{2}$ measurements. (b) Two arterioles (A1 and A2), one vein (V1), and two parenchyma regions (P1 and P2) were targeted for ${\mathrm{pO}}_{2}$ measurements after stroke induction. (c) Relative blood flow and ${\mathrm{pO}}_{2}$ within the targeted regions during and after photothrombosis. The green-shaded section indicates irradiation of the targeted arteriole. The arrow indicates the propagation of an ischemia-induced depolarization event. Scale bars=1  mm.

Targeted photothrombosis within a descending arteriole can be seen in the series of speckle contrast images in Fig. 5(a). The red overlay in the first frame depicts the $0.09\text{-}{\mathrm{mm}}^{2}$ region illuminated with DMD-targeted 532-nm light for 420 s. Because Oxyphor PtG4 has minimal absorbance of green light [Fig. 2(a)], ${\mathrm{pO}}_{2}$ measurements using 445-nm excitation were simultaneously acquired from the same region. The remaining frames depict the progression of the photothrombotic occlusion as the targeted vessel underwent stenosis and flow was significantly reduced. After 2 min of exposure, the occluded area was indistinguishable from the surrounding parenchyma.

Figure 5(b) shows the five regions targeted for continuous relative blood flow and 445-nm ${\mathrm{pO}}_{2}$ measurements. The first arteriole region (A1) is the same vessel targeted for photothrombotic occlusion. The resulting timecourses of relative blood flow and ${\mathrm{pO}}_{2}$ within each region can be seen in Fig. 5(c). By t=120  s, relative flow within the targeted arteriole decreased to <50% of baseline and ${\mathrm{pO}}_{2}$ fell from 80 to only 20 mm Hg. The propagation of an ischemia-induced depolarization event^34^^,^^35^ can be seen beginning at t=300  s with sharp reductions in both relative flow and ${\mathrm{pO}}_{2}$. As the depolarization subsided, flow within the targeted arteriole further decreased to <35% of baseline while the ${\mathrm{pO}}_{2}$ returned to predepolarization levels around 20 mm Hg.

Relative blood flow and ${\mathrm{pO}}_{2}$ decreased over the remainder of the imaging session across all regions except the targeted arteriole. At t=860  s, the vessel partially reperfused, causing a sudden increase in both relative blood flow (+6% points) and ${\mathrm{pO}}_{2}$ (+15  mm Hg). By the end of the imaging session, relative blood flow had increased to 55% of baseline and ${\mathrm{pO}}_{2}$ to 42 mm Hg, likely indicating further reperfusion of the vessel.

### 3.4 Chronic Imaging of Oxygen Tension and Blood Flow

**Fig. 6:**
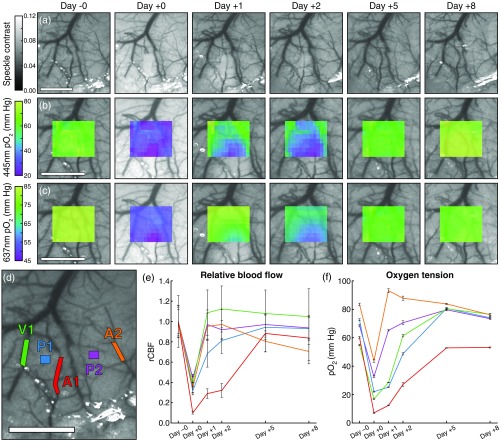
Progression of the ischemic lesion over eight days as imaged with (a) LSCI, (b) 445-nm tiled ${\mathrm{pO}}_{2}$, and (c) 637-nm tiled ${\mathrm{pO}}_{2}$ measurements. Day −0 measurements were taken immediately prior to photothrombosis induction and day +0 measurements were taken immediately after. (d) Two arterioles (A1 and A2), one vein (V1), and two parenchyma regions (P1 and P2) were targeted for chronic (e) relative blood flow and (f) 445-nm ${\mathrm{pO}}_{2}$ measurements (mean ± sd). The relative blood flow was baselined against day −0 measurements. Scale bars=1  mm.

The chronic progression of the ischemia was tracked for eight days following photothrombosis. The perfusion of the occluded arteriole and broader effect on cortical flow was tracked using LSCI as shown in Fig. 6(a). Tiled ${\mathrm{pO}}_{2}$ maps acquired using both 445- and 637-nm excitations [Figs. 6(b) and 6(c)] reveal the spatial extent of the oxygen deficit, including a large gradient between the occluded vessel and the surrounding tissue on days +1 and +2. The same five regions used during photothrombosis were targeted for chronic relative blood flow and ${\mathrm{pO}}_{2}$ measurements [Figs. 6(d)–6(f)]. Relative blood flow was calculated using the prestroke (day −0) measurements as baseline.

The first poststroke measurements (day +0) were taken immediately after the induction of photothrombosis and revealed global deficits in both blood flow and ${\mathrm{pO}}_{2}$. Over the next two days, the targeted arteriole partially reperfused and the infarct was localized to the surrounding area. By day +5, the vessel had fully reperfused and ${\mathrm{pO}}_{2}$ measurements returned to near baseline levels.

## 4 Discussion

The combination of LSCI and oxygen-dependent quenching of phosphorescence is a purely optical, noncontact strategy for measuring blood flow and oxygen tension within the cortex. This system builds upon prior work that used structured illumination to overcome traditional limitations of lifetime imaging.^18^ Spatially patterning excitation light with a DMD allows for the use of a point detector, which offers both high sensitivity and speed for the collection of the phosphorescent signal. The spatial and temporal resolutions of the system are ultimately limited by noise. Regions as small as $0.01\;{\mathrm{mm}}^{2}$ can reliably be excited at a pattern repetition rate of 10 Hz with 200 phosphorescent decays collected and averaged per pattern. Because intensity of the excitation light scales with the size of the target area, larger regions could allow for faster pattern rates at the expense of averaging (e.g., 100 Hz with 20-decay averaging). However, a 10-Hz pattern rate is more than sufficient for visualizing dynamic physiological events, such as spreading depolarizations, which have been reported to propagate at a rate of several millimeters per minute.^36^

The comparison of excitation wavelengths revealed significant differences in measured oxygen tension. Static ${\mathrm{pO}}_{2}$ measurements under 445-nm illumination spanned a range five times larger than that of 637-nm illumination. This discrepancy was most noticeable in venous regions, with the shorter excitation wavelength resulting in ${\mathrm{pO}}_{2}$ values around 50 mm Hg whereas the longer wavelength resulted in values around 75 mm Hg. Since depth penetration is heavily dependent upon wavelength,^37^ the difference is likely the result of photons sampling a much larger volume of tissue under 637-nm illumination. An estimate of transmission through a surface vessel at both wavelengths can be obtained using the Beer–Lambert law. Because scattering increases the distance traveled by photons in tissue, this represents the most conservative estimate of the effect of wavelength on transmission. Assuming hemoglobin is the primary absorber in blood plasma with a concentration of 2.3 mM^38^ and 95% ${\mathrm{SaO}}_{2}$, the transmittance at 445 and 637 nm through a 100-μm arteriole is 0.9% and 96.5%, respectively. As excitation wavelength approaches the tissue optical window, the transmission of incident light significantly increases. The 445-nm light is almost entirely confined within a vessel of that caliber and does not extensively sample deeper microvasculature. Because the 637-nm light penetrates further into the brain, the measured ${\mathrm{pO}}_{2}$ is skewed away from the vascular value and is more representative of a bulk volumetric average. This is consistent with prior Monte Carlo modeling that found fluorescence primarily originates from within large surface vasculature at shorter wavelengths.^20^ As excitation wavelength increases, a larger fraction of the detected fluorescence originates from beyond the vessel.

The creation of an extended occlusion within an arteriole using targeted photothrombosis was demonstrated for the first time. Previous methods relied upon broad illumination to occlude a large volume of vasculature^30^ or highly focused light to occlude a single part of a microvessel.^39^ The previous iteration of this system could only induce occlusions within a large region, and ${\mathrm{pO}}_{2}$ measurements could not be simultaneously acquired.^18^ While photothrombosis is widely utilized, there is evidence it does not produce pathophysiologically relevant ischemic lesions.^40^ This system allows for greater control over the spatial characteristics of the stroke (e.g., size and location) and the option to target the entirety of individual vessels or even multiple vessels simultaneously.

While tissue ${\mathrm{pO}}_{2}$ during ischemic depolarizations has been previously examined,^41^ to our knowledge, no studies have quantified the acute vascular response to a depolarization event or the chronic response to an ischemic infarct. The depolarization results in a global flow reduction across all regions, which is consistent with previous reports using other stroke models.^34^^,^^42^ Within the targeted arteriole, the blood flow reduction is of greater magnitude (−58%) than the corresponding decrease in ${\mathrm{pO}}_{2}$ (−44%), which eventually recovers to slightly above predepolarization levels. Unfortunately, it is difficult to predict how the ${\mathrm{pO}}_{2}$ responded to the depolarization across the other regions, but it likely mirrored the LSCI results.

The chronic measurements revealed a severe poststroke blood flow and oxygen deficit that recovered almost completely within five days. The spatial extent of the ischemia can be clearly seen on days +1 and +2 in both the speckle contrast imagery and tiled ${\mathrm{pO}}_{2}$ maps in Fig. 6. The large gradient between the occluded vessel and surrounding tissue resembles the ischemic penumbra that the photothrombotic technique rarely produces.^40^ By patterning illumination light instead of irradiating a circular region, the photothrombosis is contained within the targeted vessel, which allows the infarct to manifest downstream of the occlusion. By day +5, the targeted vessel appeared to have fully reperfused and there was no evidence of hypoxia in the tiled ${\mathrm{pO}}_{2}$ measurements. The speed of this recovery is faster than previously reported^43^ but can likely be explained by the smaller area targeted for photothrombosis, which resulted in a less severe ischemic lesion.

### 4.1 Limitations

A potential concern for this system is the scattering of light beyond the desired region of interest when performing the ${\mathrm{pO}}_{2}$ measurements or targeted photothrombosis. This problem is highlighted by the discrepancies seen in ${\mathrm{pO}}_{2}$ values under 445- and 637-nm illuminations and introduces the risk of collateral tissue damage. The wavelength dependence of light propagation also means that the LSCI and ${\mathrm{pO}}_{2}$ measurements sample different volumes of tissue. The use of anesthesia during imaging significantly affects systemic hemodynamics^44^ and has been shown to inhibit the oxygen autoregulatory response.^45^ Because the animals are used for chronic imaging, tracheal intubation for mechanical ventilation is not an option. This may introduce inconsistencies in breathing that can skew both blood flow and ${\mathrm{pO}}_{2}$ measurements. The implementation of an awake imaging setup^46^ would resolve this issue by completely eliminating the need for anesthesia during imaging. Another concern is that estimates of blood flow provided by single-exposure speckle imaging have limitations for chronic imaging or cross-animal comparisons.^47^ An enhanced technique known as multiexposure speckle imaging (MESI) has been developed to increase the quantitative accuracy of flow measurements.^48^ Implementing MESI would allow for more robust chronic measurements of blood flow in the ischemic brain.

## 5 Conclusion

We have presented an imaging system capable of simultaneously measuring relative cerebral blood flow and vascular oxygen tension at higher spatiotemporal resolutions than its predecessor. We demonstrated the ability to perform targeted ${\mathrm{pO}}_{2}$ measurements *in vivo* using both 445- and 637-nm illuminations. The discrepancy in ${\mathrm{pO}}_{2}$ values between the two wavelengths highlighted the influence of penetration depth on single-photon phosphorescence measurements. We also demonstrated the induction of a DMD-targeted photothrombotic stroke within a single vessel and imaged blood flow and oxygen tension during a subsequent ischemic depolarization. Chronic imaging revealed a rapid recovery with the occluded vessel fully reperfusing and the ${\mathrm{pO}}_{2}$ returning to baseline levels within only five days. This system will have broad applications for studying the progression of ischemic stroke and other vascular pathologies in the brain.
